# Circuits in the Ventral Medulla That Phase-Lock Motoneurons for Coordinated Sniffing and Whisking

**DOI:** 10.1155/2016/7493048

**Published:** 2016-05-18

**Authors:** Martin Deschênes, Anastasia Kurnikova, Michael Elbaz, David Kleinfeld

**Affiliations:** ^1^Department of Psychiatry and Neuroscience, Laval University, Québec City, QC, Canada G1J 2R3; ^2^Section of Neurobiology, University of California, San Diego, CA 92093, USA; ^3^Department of Physics, University of California, San Diego, CA 92093, USA; ^4^Department of Electrical and Computer Engineering, University of California, San Diego, CA 92093, USA

## Abstract

The exploratory behavior of rodents is characterized by stereotypical movements of the vibrissae, nose, and head, which are phase locked with rapid respiration, that is, sniffing. Here we review the brainstem circuitry that coordinates these actions and propose that respiration may act as a master clock for binding orofacial inputs across different sensory modalities.

## 1. Introduction

**Figure 1:**
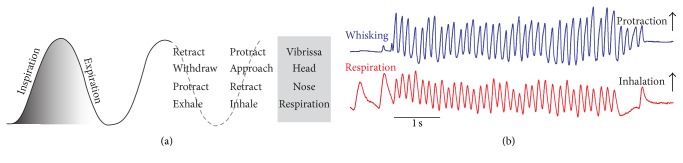
Cooccurrence of sniffing and whisking in rodents. (a) Sniff and whisk cycles are coordinated with nose and head movements (adapted from Welker [1]). (b) The motion of vibrissa D2 was monitored by high-speed videography (250 frames per second), and sniffing was recorded by means of a thermocouple implanted in the nasal cavity. In all figures inspiration and vibrissa protraction are up.

When one observes rodents introduced in a new environment, one immediately notices that they are extremely curious. They run about, stand up on their hind legs, crane their necks forward, and explore the environment by sniffing and whisking vigorously. In a classic paper published in 1964 [1], Welker provided the first descriptive account of the sniffing behavior in rats. Using cinematographic technique, he reported that sniffing consists of an integrated sequence of movements that involve (1) bursts of polypnea; (2) recurrent protraction and retraction of mystacial vibrissae; (3) repetitive retraction and protraction of the tip of the snout; and (4) a rapid series of head movements and fixations. These four components occur at rates between 5 and 11 Hz, in bouts of one to many seconds, and “exhibit a fixed temporal relationship to one another” (Welker [1]). This temporal relationship is illustrated in Figure 1, where whisking and sniffing were recorded as a rat explores the exit of a tunnel.

The coordination of whisking, head bobbing, and nose motion with sniffing requires a hierarchal organization of the brainstem circuitry so that the occurrence and timing of these actions do not interfere with each other and with fundamental metabolic needs. Here we review evidence for phase locking of rhythmic orofacial actions with breathing, particularly in the context of the synaptic mechanisms that coordinate sniffing, whisking, and nose motion, which are predominant activities during exploratory behaviors in rodents [2, 3].

## 2. Facial Muscles and Their Central Representation

**Figure 2:**
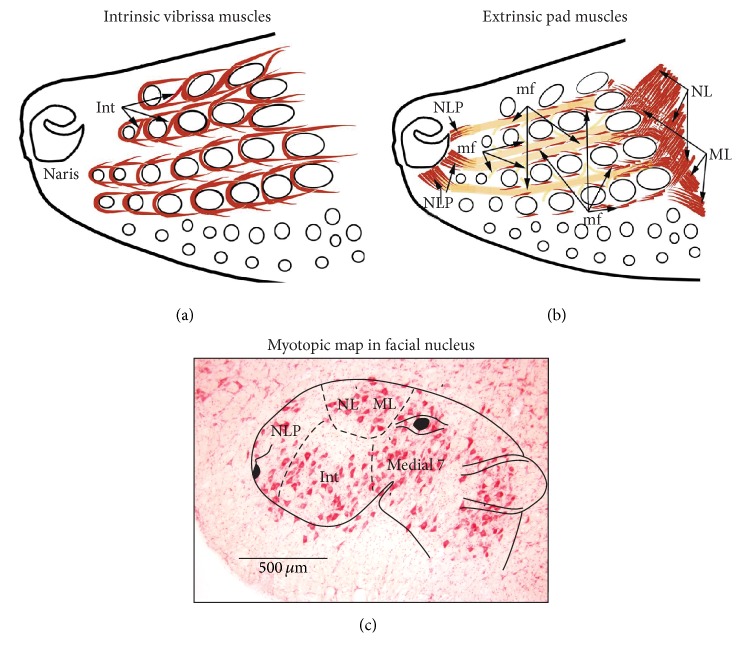
Facial muscles involved in exploratory behavior in rodents. (a) The intrinsic muscles (Int) form a sling around the base of vibrissa follicles. When they contract, vibrissae protract. (b) Two groups of extrinsic muscles translate the mystacial pad: muscle nasolabialis (NL) and maxillolabialis (ML) retract the pad, while muscle nasolabialis profundus (NLP) protracts the pad. Extrinsic muscle fibers (mf) run underneath the skin between vibrissa rows. (c) Motoneurons that innervate the intrinsic vibrissa muscles are located in the ventral lateral part of the facial nucleus, the extrinsic retractor muscles (nasolabialis and maxillolabialis) are represented dorsolaterally, and the extrinsic protractor muscles (nasolabialis profundus) are represented at the lateral most edge of the nucleus. The drawings in (a) and (b) were adapted from Figure  11 of Grant et al. [15].

Nasofacial muscles of rodents that control movement of the vibrissae, the opening of the nares, and deflection of the nose are innervated by facial motoneurons [4–6]. Several studies have shown that facial motoneurons in rodents are functionally organized into clusters [7–14].  Figure 2 shows a schematic representation of the facial muscles and the corresponding myotopic map in the facial nucleus. Motoneurons that innervate the intrinsic vibrissa muscles that protract the vibrissae are located in the ventral lateral part of the nucleus; the extrinsic retractor muscles (nasolabialis and maxillolabialis) that translate the mystacial pad in the caudal direction are represented dorsolaterally; the extrinsic protractor muscles (nasolabialis profundus) that translate the pad rostrally and open the nares are represented at the lateral edge of the nucleus. Muscle deflector nasi, which is not shown in the drawings of Figure 2 (but see Figure 5(a)), is represented dorsolaterally.

Studies using transsynaptic retrograde labeling have allowed the identification of premotor neurons that control each of these muscle groups [13, 14]. We thus focus next on how rhythmic activity of these different pools of premotor neurons coordinates with respiration.

## 3. Rhythmogenesis of Sniffing and Whisking

Sniffing in rats is usually defined as rapid, rhythmic respiration [16, 17]. Rats, unlike humans, always breathe nasally; lower frequencies (1–3 Hz) correspond to basal respiration and higher frequencies (4–12 Hz) correspond to sniffing. It is now well established that a medullary region called the pre-Bötzinger complex (preBötC) forms the core of the respiratory generator (see review by Feldman and Kam [18]) and that sniffing is associated with an increased rate of bursts in preBötC cells [19]. The neuronal circuits that accelerate the respiratory rhythm upon presentation of an odorant remain unknown.

Welker [1] was the first to propose that a brainstem oscillator drives whisking. This hypothesis derived from the observation that whisking persists after motor cortex ablation and that bilateral section of the infraorbital nerve, that is, sensory denervation, has little effect on the generation, kinematics, and bilateral coordination of the normal whisking pattern. Recently the whisking oscillator was discovered in a medullary region close to the preBötC [19].

## 4. Premotor Circuits for Sniffing

**Figure 3:**
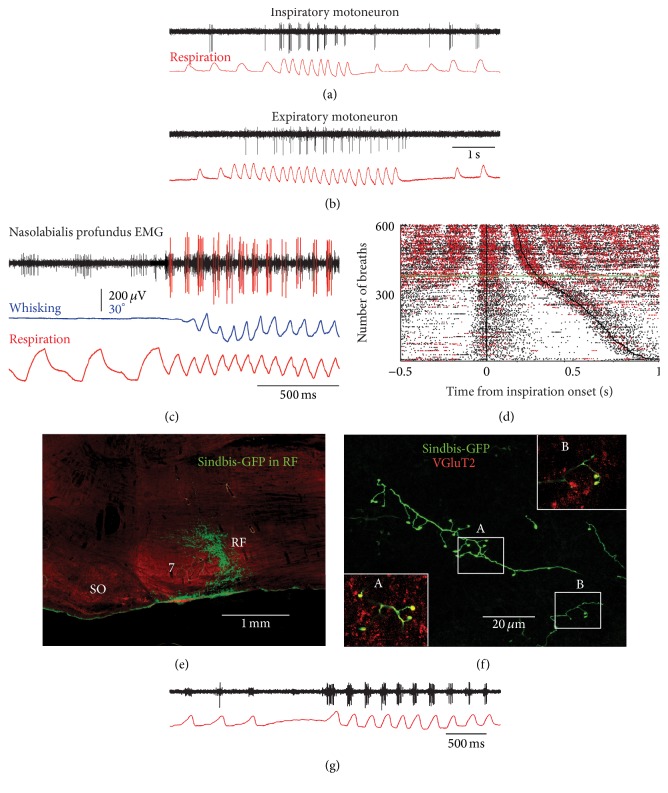
Facial motoneurons and sniffing. (a, b) Recording of facial motoneurons in alert head-restrained rats reveals respiration-related cells that fire preferentially during sniffing. (c) Electromyographic recording of NLP motor units during basal respiration and sniffing. Note that the small unit is active during both basal respiration and sniffing, while the large unit only fires during sniffing. (d) Raster plots of the activity of NLP motor units relative to the onset of inspiration (black lines). Black and red dots represent spikes of the small and large motor units shown in (c), respectively. Individual breaths are ordered by the duration of the breath. The green line indicates the transition between basal respiration and sniffing. Note that the small unit is active during inspiration at all breathing frequencies, while the large unit is preferentially active during sniffing. (e) Sindbis-GFP injection in the retrofacial region leads to anterograde labeling in the facial nucleus. (f) Confocal microscopy reveals a majority of labeled boutons that are immunopositive for type 2 vesicular glutamate transporter (VGluT2). Framed areas A and B are enlarged in the corresponding inserts. (g) Example of respiration-related neurons of the retrofacial region that are principally recruited during sniffing. Data in panels (a) to (d) are adapted from [20], while data in panels (e) to (g) are unpublished results.

Transsynaptic labeling studies demonstrated that the preBötC and the parafacial respiratory region project to the facial nucleus [13, 14]. Neuronal and electromyographic (EMG) recordings in alert rats further showed that motoneurons that innervate the extrinsic muscles, that is, muscles nasolabialis, maxillolabialis, and nasolabialis profundus, discharge in a phase-locked manner with the respiratory cycle [19]. Yet, most of these muscles are differentially active during basal respiration versus sniffing (Figures 3(a)–3(d)). As a case in point, muscle nasolabialis profundus has two components. One attaches to the plate of the mystacial pad (pars* maxillaris*), which contributes to dilation of the nares [21], and another one that attaches to the corium (*pars media*) that translates the mystacial pad rostrally [5, 6, 22]. Both muscle components contract at the onset of inspiration. While naris dilation occurs with each inspiration in awake rats, rostral translation of the pad occurs primarily when the rate or amplitude of respiration increases [20, 22]. The same is true for the extrinsic retractor muscles (nasolabialis and maxillolabialis) and the nasi deflector muscle, which are principally active when the animal sniffs or whisks [22, 23]. The preferential recruitment of these muscles during sniffing could depend on modulatory action that enhances the excitability of the motoneurons* per se* or of the associated premotor circuits.

By and large, preBötC cells that constitute the respiratory pattern generator are glutamatergic and express somatostatin or the neurokinin-1 receptor. Interestingly, Takatoh et al. [13] reported that few preBötC cells labeled after ΔG-rabies injection in the mystacial pad express these phenotypes. Furthermore, injection of an adeno-associated virus that expresses eGFP driven by the somatostatin promoter in the preBötC did not label terminal fields in the facial nucleus [24]. Since the deletion or silencing of neurokinin-1 receptor and somatostatin-expressing preBötC neurons, respectively, disrupts breathing in the adult rat [25, 26], the preBötC projection to facial motoneurons may arise from cells that are not themselves part of the respiratory rhythm generator. The latter cells may be recruited when the animal sniffs, or the conditional respiratory drive may involve follower neurons intercalated between the preBötC and facial motoneurons. The region immediately caudal to the facial nucleus, referred to here as the retrofacial region, appears as a potential source of this conditional drive. It receives input from the preBötC and contains glutamatergic premotor neurons (Figures 3(e) and 3(f)). Exploratory recordings in this region revealed a number of neurons that are recruited when the breathing rhythm accelerates (Figure 3(g)). However, it remains unclear whether these cells are part of the parafacial respiratory group as delineated in prior studies [27–29].

## 5. Premotor Circuits for Whisking

**Figure 4:**
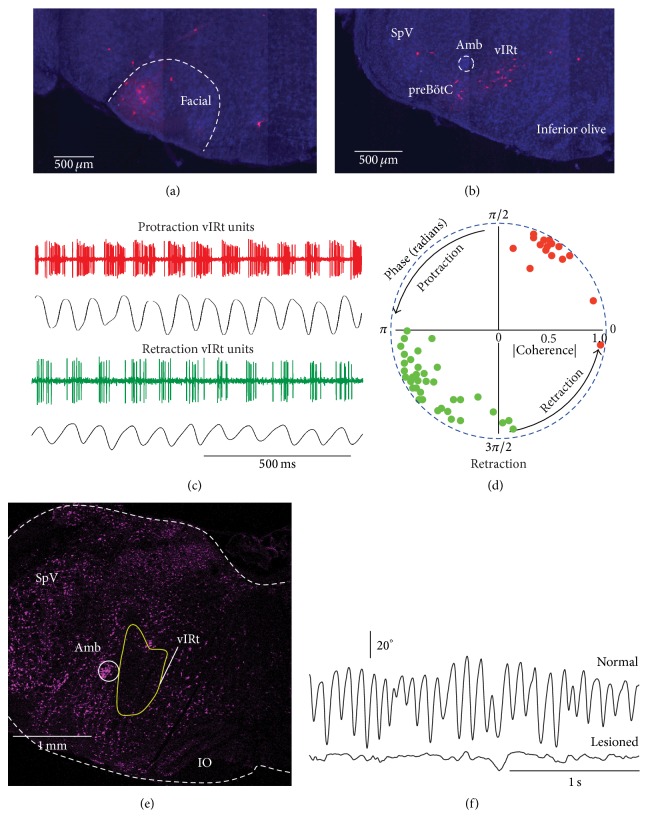
Facial motoneurons and whisking. (a) ΔG-rabies injection into the mystacial pad in mice label motoneurons in the ventral lateral part of the facial nucleus, and premotor neurons (b) in the intermediate reticular formation of the medulla (vIRt). (c) vIRt cells discharge in a phase-locked manner with vibrissa motion during whisking induced by kainic acid injection in the medulla. (d) Polar plot of the coherence between spiking activity of vIRt cells and vibrissa motion at the peak frequency of whisking. Red and green dots represent protraction units and retraction units, respectively. Over 200 whisks per cell were used to compute phase angle and coherence. (e) Ibotenic acid lesion of vIRt leads to abolition of whisking on the side of the lesion (f). All panels are adapted from [19].

Premotor neurons that generate whisking are located in the vibrissa-related region of the intermediate reticular formation (vIRt) of the medulla adjacent to the preBötC [19] (Figures 4(a) and 4(b)). This proximity appears functionally relevant since whisking is tightly coupled to sniffing, yet it is separately gated [1, 19, 30]. Cells in the vIRt fire either in phase or in antiphase with whisker protraction (Figures 4(c) and 4(d)), selective lesion of the vIRt abolishes whisking on the side of the lesion (Figures 4(e) and 4(f)), and activation of the vIRt by iontophoretic injection of kainic acid induces long periods of continuous whisking in the lightly anesthetized rat [31]. Together, these results indicate that the vIRt is both necessary and sufficient to generate whisking.

It was recently shown that glycinergic vIRt cells are key elements in whisking generation [20]. This finding is consistent with the notion that sustained depolarizing inputs to the facial motoneurons determine the maximum protraction of the vibrissae, while the whisking oscillator rhythmically inhibits these motoneurons. Thus, the vIRt oscillator drives whisking on the retraction phase, opposite from what was long supposed. The inhibitory nature of the whisking oscillator calls for a reappraisal of the control of brainstem circuits by top-down inputs for the control of amplitude, frequency, and set-point of whisking.

Phase sensitivity analysis of coupling between whisking and breathing has shown that inspiration can reset whisking, but not vice versa [19]. Unidirectional phase resetting of whisking by basal respiration and sniffing is mediated by unidirectional connections from the preBötC to the vIRt [20].

Natural whisking is bilaterally synchronous in the absence of external objects or head turning [32–34]. This requires a circuitry that coordinates the activity of the left and right whisking oscillators. Yet, tract tracing by means of conventional tracers or virus injection did not reveal connections between the left and right vIRts [20]. Moreover, spectral analysis revealed that the two whisking oscillators begin to drift in phase when respiration is transiently halted during a sigh or during apnea induced by inhalation of ammonia vapor [20]. This is consistent with the notion that the left and right whisking oscillators are independent from one another and therefore begin to drift in the absence of repeated resetting events. Together these results indicate that either commissural connections between vIRTs are absent or, if present, they are not strong enough to synchronize whisking bilaterally. Alternatively, several studies have established that glutamatergic preBötC cells are interconnected by commissural axons [35–37]. Impairment of these connections in Robo knockout mice leads to desynchronization of the excitatory drive to the left and right spinal and cranial motoneurons [38]. Given that each preBötC projects to vIRt, commissural connections between the respiratory pattern generators represent the most likely substrate for the bilateral synchronization of whisking.

Several behavioral studies have reported that the degree of phase coupling between whisking on both sides of the face or between whisking and sniffing depends on context, motor strategies, and sensory feedback [33, 34, 39–41]. This indicates that, like other central pattern generators, the whisking oscillator can be modulated to adapt to the organism's circumstances and needs [42, 43]. For the moment, brainstem and top-down inputs that control the vIRt and the coupling between the vIRt and the preBötC remain unknown.

## 6. Premotor Circuits for Nose Motion

**Figure 5:**
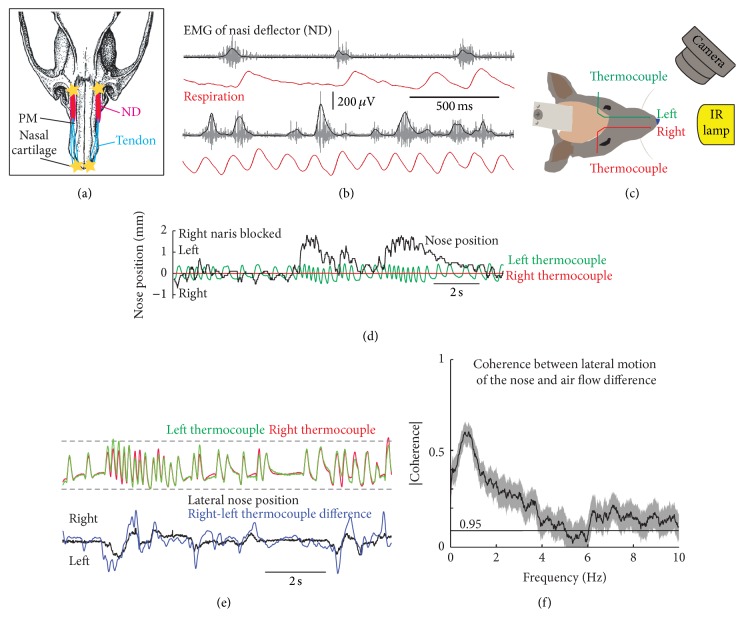
Facial motoneurons and nose motion. (a) Muscle nasi deflector (ND) is located laterally to the premaxillary bone (PM). It takes origin at the orbital edge of the maxilla and its long tendon inserts over the nasal cartilage (stars, attachment sites). (b) Electromyographic recordings in alert head-restrained rats reveal that muscle ND is active during the late phase of expiration during basal respiration (upper traces) and during the early phase of expiration during sniffing (lower traces); black traces, smoothed rectified EMGs. (c) Experimental setup to measure nose motion and nasal airflow in head-restrained rats; IR, infrared light. (d) Blockade of the right naris with a polymer compound abolishes respiratory signals from the right thermocouple and biases nose deflection towards the left side of the face. (e) Upper traces show normalized respiratory signals recorded from the left and right nostrils. Lower traces show that change in lateral nose position is associated with change in airflow through the left and right nostrils as estimated from difference in the amplitude of thermocouple signals. (f) Spectral coherence between lateral displacement of the nose and air flow difference between the left and right nostrils. Black line, 95% confidence level based on Gaussian approximation; light red area, 95% confidence interval based on boot-strap. All data in this figure are unpublished results.

A seldom-studied aspect of sniffing is nose motion, which is particularly noticeable in high-speed video recordings. The nose of rodents is made of cartilages that form a telescopic connection with the nasal and premaxillary bones and permits bending of the nose in the dorsoventral and lateral directions [6]. Nose movements are controlled by muscle deflector nasi, which attaches to the orbital edge of the maxillary bone and to the aponeurosis above nasal cartilages (Figure 5(a)). Bilateral contraction of this muscle raises the tip of the nose, while unilateral contraction produces lateral deflection [21]. EMG recordings in head-restrained rats reveal that muscle deflector nasi contracts during the late phase of expiration during basal respiration and shifts to early expiration during sniffing (Figure 5(b)), which allows repositioning of the nares for the next inhalation. Furthermore, monitoring nasal airflow through each of the nares reveals that nose deflection is associated with difference in airflow in each of the nares (Figures 5(c)–5(e)). Rabies injection into muscle nasi deflector leads to transsynaptic labeling in the retrofacial region (unpublished data), but physiological evidence that retrofacial premotor neurons actually control nose motion is lacking.

## 7. The Respiratory Oscillator as a Master Clock

The observation of precise phase locking between sniffing and exploratory whisking leads to the hypothesis that the breathing rhythm functions as the reference oscillation for the alignment of commensurate signals [2]. For example, when rodents are actively exploring their environment, phase locking between whisking and sniffing could ensure that spikes induced by tactile and olfactory stimuli occur with a fixed temporal relationship to one another, which corresponds to an object with a particular smell at a particular location relative to the face. This could provide a means to bind olfactory inputs, which enter the brain at its rostral pole, with coincident inputs from touch, which enter the brain at the level of the brainstem. It obviates the need for a direct neuronal projection of respiratory output between these two regions. This scheme may be readily extended to taste through the entrainment of licking and thus covers the full range of stimuli required to assess the shape, odor, texture, and taste of food.

## 8. Why Do Rodents Sniff and Whisk?

Although sniffing clearly serves olfaction and strongly patterns olfactory processing, several studies reported that basal respiratory rhythm is sufficient for delivering odorants to olfactory receptors and that rats and mice can perform simple odor discrimination after a single sniff [17, 44, 45]. Likewise, rodents can locate objects and gauge aperture widths with a single whisk, and severing the facial nerve to block whisking does not affect performance [46, 47]. Lastly, rodents can perform many tactile tasks without whisking* per se*, either by using head and body movements to move the vibrissae or by maintaining their vibrissae still in a region of interest where contact is expected [47].

Given the above evidence that olfaction can occur without sniffing and vibrissa touch can be effective without whisking, the questions remain as to why rodents sniff, whisk, and move their nose. It is intriguing that rats deprived of olfactory and vibrissa afferents continue to sniff and whisk in a relatively normal manner [1]. We suggest three reasons as to why rhythmic activity is associated with the use of these sensors. First, as rodents have relatively limited visual capabilities and are the targets of predators, fast sampling of the immediate environment has clear survival value [46]. The few extra hundred milliseconds of warning that is gained by scanning the environment may be sufficient for escape. Second, sniffing and whisking not only serve odor sampling and touch, but also constitute activity patterns that are the overt expression of reward expectation [48–50]. Perhaps not independent of this, sniffing is commonly displayed during motivated and social behaviors to communicate and convey information about social hierarchy [51]. Finally, the frequency of whisking and sniffing lies close to that of the hippocampal theta rhythm. These rhythms are incommensurate during foraging and exploration [52], yet they lock for a brief epoch during the approach to a stimulus during forced choice tasks [53, 54]. Thus, rhythmic dynamics may serve to facilitate transient coherence between two regions of the brain [55]. From this standpoint, respiration is more than a rhythm for life, but it also coordinates orofacial motor commands that engage common muscle groups, serves a variety of active sensory and social behaviors, and may coordinate sensory input with memory formation and recall.

## References

[1] Welker W. (1964). Analysis of sniffing of the albino rat. *Behaviour*.

[2] Kleinfeld D., Deschênes M., Wang F., Moore J. D. (2014). More than a rhythm of life: breathing as a binder of orofacial sensation. *Nature Neuroscience*.

[3] Deschênes M., Moore J., Kleinfeld D. (2012). Sniffing and whisking in rodents. *Current Opinion in Neurobiology*.

[4] Haidarliu S., Simony E., Golomb D., Ahissar E. (2010). Muscle architecture in the mystacial pad of the rat. *Anatomical Record*.

[5] Haidarliu S., Golomb D., Kleinfeld D., Ahissar E. (2012). Dorsorostral snout muscles in the rat subserve coordinated movement for whisking and sniffing. *Anatomical Record*.

[6] Haidarliu S., Kleinfeld D., Deschênes M., Ahissar E. (2015). The musculature that drives active touch by vibrissae and nose in mice. *Anatomical Record*.

[7] Watson C. R. R., Sakai S., Armstrong W. (1982). Organization of the facial nucleus in the rat. *Brain, Behavior and Evolution*.

[8] Ashwell K. W. (1982). The adult mouse facial nerve nucleus: morphology and musculotopic organization. *Journal of Anatomy*.

[9] Hinrichsen C. F. L., Watson C. D. (1984). The facial nucleus of the rat: representation of facial muscles revealed by retrograde transport of horseradish peroxidase. *Anatomical Record*.

[10] Komiyama M., Shibata H., Suzuki T. (1984). Somatotopic representation of facial muscles within the facial nucleus of the mouse: a study using the retrograde horseradish peroxidase and cell degeneration techniques. *Brain, Behavior and Evolution*.

[11] Furutani R., Izawa T., Sugita S. (2004). Distribution of facial motoneurons innervating the common facial muscles of the rabbit and rat. *Okajimas Folia Anatomica Japonica*.

[12] Klein B. G., Rhoades R. W. (1985). Representation of whisker follicle intrinsic musculature in the facial motor nucleus of the rat. *Journal of Comparative Neurology*.

[13] Takatoh J., Nelson A., Zhou X. (2013). New modules are added to vibrissal premotor circuitry with the emergence of exploratory whisking. *Neuron*.

[14] Sreenivasan V., Karmakar K., Rijli F. M., Petersen C. C. H. (2015). Parallel pathways from motor and somatosensory cortex for controlling whisker movements in mice. *European Journal of Neuroscience*.

[15] Grant R. A., Haidarliu S., Kennerley N. J., Prescott T. J. (2013). The evolution of active vibrissal sensing in mammals: evidence from vibrissal musculature and function in the marsupial opossum *Monodelphis domestica*. *Journal of Experimental Biology*.

[16] Youngentob S. L., Mozell M. M., Sheehe P. R., Hornung D. E. (1987). A quantitative analysis of sniffing strategies in rats performing odor detection tasks. *Physiology and Behavior*.

[17] Uchida N., Mainen Z. F. (2003). Speed and accuracy of olfactory discrimination in the rat. *Nature Neuroscience*.

[18] Feldman J. L., Kam K. (2015). Facing the challenge of mammalian neural microcircuits: taking a few breaths may help. *Journal of Physiology*.

[19] Moore J. D., Deschênes M., Furuta T. (2013). Hierarchy of orofacial rhythms revealed through whisking and breathing. *Nature*.

[20] Deschênes M., Takatoh J., Kurnikova A. (2016). Inhibition, not excitation, drives rhythmic whisking. *Neuron*.

[21] Deschênes M., Haidarliu S., Demers M., Moore J., Kleinfeld D., Ahissar E. (2015). Muscles involved in naris dilation and nose motion in rat. *Anatomical Record*.

[22] Hill D. N., Bermejo R., Zeigler H. P., Kleinfeld D. (2008). Biomechanics of the vibrissa motor plant in rat: rhythmic whisking consists of triphasic neuromuscular activity. *The Journal of Neuroscience*.

[23] Sherrey J. H., Megirian D. (1977). State dependence of upper airway respiratory motoneurons: functions of the cricothyroid and nasolabial muscles of the unanesthetized rat. *Electroencephalography and Clinical Neurophysiology*.

[24] Tan W., Pagliardini S., Yang P., Janczewski W. A., Feldman J. L. (2010). Projections of preBötzinger complex neurons in adult rats. *Journal of Comparative Neurology*.

[25] Gray P. A., Janczewski W. A., Mellen N., McCrimmon D. R., Feldman J. L. (2001). Normal breathing requires preBötzinger complex neurokinin-1 receptor-expressing neurons. *Nature Neuroscience*.

[26] Tan W., Janczewski W. A., Yang P., Shao X. M., Callaway E. M., Feldman J. L. (2008). Silencing preBötzinger complex somatostatin-expressing neurons induces persistent apnea in awake rat. *Nature Neuroscience*.

[27] Fortuna M. G., West G. H., Stornetta R. L., Guyenet P. G. (2008). Bötzinger expiratory-augmenting neurons and the parafacial respiratory group. *The Journal of Neuroscience*.

[28] Abbott S. B. G., Stornetta R. L., Coates M. B., Guyenet P. G. (2011). Phox2b-expressing neurons of the parafacial region regulate breathing rate, inspiration, and expiration in conscious rats. *The Journal of Neuroscience*.

[29] Pagliardini S., Janczewski W. A., Tan W., Dickson C. T., Deisseroth K., Feldman J. L. (2011). Active expiration induced by excitation of ventral medulla in adult anesthetized rats. *The Journal of Neuroscience*.

[30] Ranade S., Hangy B., Kepecs A. (2013). Multiple modes of phase locking between sniffing and whisking during active exploration. *The Journal of Neuroscience*.

[31] Moore J. D., Deschênes M., Kurnikova A., Kleinfeld D. (2014). Activation and measurement of free whisking in the lightly anesthetized rodent. *Nature Protocols*.

[32] Gao P., Hattox A. M., Jones L. M., Keller A., Zeigler H. P. (2003). Whisker motor cortex ablation and whisker movement patterns. *Somatosensory and Motor Research*.

[33] Towal R. B., Hartmann M. J. (2006). Right-left asymmetries in the whisking behavior of rats anticipate head movements. *The Journal of Neuroscience*.

[34] Mitchinson B., Martin C. J., Grant R. A., Prescott T. J. (2007). Feedback control in active sensing: rat exploratory whisking is modulated by environmental contact. *Proceedings of the Royal Society B: Biological Sciences*.

[35] Wang H., Stornetta R. L., Rosin D. L., Guyenet P. G. (2001). Neurokinin-1 receptor-immunoreactive neurons of the ventral respiratory group in the rat. *Journal of Comparative Neurology*.

[36] Stornetta R. L., Rosin D. L., Wang H., Sevigny C. P., Weston M. C., Guyenet P. G. (2003). A group of glutamatergic interneurons expressing high levels of both neurokinin-1 receptors and somatostatin identifies the region of the pre-Bötzinger complex. *Journal of Comparative Neurology*.

[37] Koizumi H., Koshiya N., Chia J. X. (2013). Structural-functional properties of identified excitatory and inhibitory interneurons within pre-Bötzinger complex respiratory microcircuits. *The Journal of Neuroscience*.

[38] Bouvier J., Thoby-Brisson M., Renier N. (2010). Hindbrain interneurons and axon guidance signaling critical for breathing. *Nature Neuroscience*.

[39] Carvell G. E., Simons D. J. (1990). Biometric analyses of vibrissal tactile discrimination in the rat. *The Journal of Neuroscience*.

[40] Cao Y., Roy S., Sachdev R. N. S., Heck D. H. (2012). Dynamic correlation between Whisking and breathing rhythms in mice. *The Journal of Neuroscience*.

[41] Fonio E., Gordon G., Barak N. (2016). Coordination of sniffing and whisking depends on the mode of interaction with the environment. *Israel Journal of Ecology & Evolution*.

[42] Ramirez J.-M., Tryba A. K., Peña F. (2004). Pacemaker neurons and neuronal networks: an integrative view. *Current Opinion in Neurobiology*.

[43] Marder E. (2012). Neuromodulation of neuronal circuits: back to the future. *Neuron*.

[44] Kepecs A., Uchida N., Mainen Z. F. (2007). Rapid and precise control of sniffing during olfactory discrimination in rats. *Journal of Neurophysiology*.

[45] Wachowiak M. (2011). All in a sniff: olfaction as a model for active sensing. *Neuron*.

[46] Krupa D. J., Matell M. S., Brisben A. J., Oliveira L. M., Nicolelis M. A. L. (2001). Behavioral properties of the trigeminal somatosensory system in rats performing whisker-dependent tactile discriminations. *The Journal of Neuroscience*.

[47] O'Connor D. H., Peron S. P., Huber D., Svoboda K. (2010). Neural activity in barrel cortex underlying vibrissa-based object localization in mice. *Neuron*.

[48] Clarke S., Trowill J. A. (1971). Sniffing and motivated behavior in the rat. *Physiology and Behavior*.

[49] Richard Waranch H., Terman M. (1975). Control of the rat's sniffing behavior by response-independent and dependent schedules of reinforcing brain stimulation. *Physiology and Behavior*.

[50] Ikemoto S., Panksepp J. (1994). The relationship between self-stimulation and sniffing in rats: does a common brain system mediate these behaviors?. *Behavioural Brain Research*.

[51] Wesson D. W. (2013). Sniffing behavior communicates social hierarchy. *Current Biology*.

[52] Berg R. W., Whitmer D., Kleinfeld D. (2006). Exploratory whisking by rat is not phase-locked to the hippocampal theta rhythm. *Journal of Neuroscience*.

[53] Macrides F., Eichenbaum H. B., Forbes W. B. (1982). Temporal relationship between sniffing and the limbic *θ* rhythm during odor discriminatin reversal learning. *The Journal of Neuroscience*.

[54] Grion N., Akrami A., Zuo Y., Stella F., Diamond M. E., Petersen C. C. (2016). Coherence between rat sensorimotor system and hippocampus is enhanced during tactile discrimination. *PLoS Biology*.

[55] Kleinfeld D., Deschênes M., Ulanovsky N. (2016). Whisking, sniffing, and the hippocampal *θ*-rhythm: a tale of two oscillators. *PLoS Biology*.

